# Prevalence and prognostic role of hypochloremia in patients with acute heart failure in Ethiopia: A single-center retrospective analysis

**DOI:** 10.1371/journal.pone.0310251

**Published:** 2024-09-12

**Authors:** Gashaw Solela

**Affiliations:** Department of Internal Medicine, Yekatit 12 Hospital Medical College, Addis Ababa, Ethiopia; Federal Medical Centre Umuahia, NIGERIA

## Abstract

**Background:**

In patients with heart failure (HF), multiple electrolyte disorders are common, and recent studies have shown that chloride disorders play a significant role in the prognosis of HF. Data about the prevalence and prognostic role of hypochloremia in patients with acute HF (AHF) are scarce in African nations, including Ethiopia. Hence, this study aimed to assess the prevalence, associated factors, and prognostic role of hypochloremia in patients with AHF in Ethiopia.

**Methods:**

This was a single-center retrospective analysis of AHF patients, aged ≥15 years, with chloride determination upon admission to the medical wards and medical ICU of Yekatit 12 Hospital Medical College, Addis Ababa, Ethiopia, from July 1, 2022, to July 1, 2023. Statistical Package for Social Sciences, version 26, was used to enter and analyze the data. Descriptive analysis was used to summarize clinical profiles, laboratory data, and outcomes of AHF patients stratified by the presence and absence of hypochloremia. Logistic regression analysis was used to determine the factors associated with hypochloremia and to assess the association of hypochloremia and other factors with in-hospital mortality in patients with AHF. A two-tailed *P* value <0.05 was regarded as statistically significant.

**Results:**

A total of 267 AHF patients who had chloride determination on admission were included in this study; the mean age was 56.7 years (standard deviation: 18.6), and the gender-based distribution of the patients was nearly equal. The prevalence of hypochloremia was 36.7%. Diastolic blood pressure <60 mm Hg [adjusted odds ratio (AOR) = 3.63, 95% confidence interval (CI), 1.04, 12.72] and hyponatremia (AOR = 29.20, 95% CI, 13.21, 64.56) were significantly associated with hypochloremia. The in-hospital mortality rate was higher in AHF patients with hypochloremia (16.3%) compared to those without hypochloremia (4.7%). The odds of in-hospital mortality among AHF patients with hypochloremia were 2.8 times higher compared to patients without hypochloremia (AOR = 2.82, 95% CI: 1.08, 7.04) after adjusting for ICU admission, systolic blood pressure < 120 mm Hg and diastolic blood pressure < 60 mm Hg.

**Conclusions:**

This study revealed a high prevalence of hypochloremia among patients with AHF. Low diastolic blood pressure and hyponatremia were significantly associated with the development of hypochloremia. Most importantly, AHF patients with hypochloremia had a higher in-hospital mortality rate compared to those without hypochloremia. Hence, hypochloremia on admission should be considered a potential prognostic factor in patients with AHF, and further research with a larger sample size is needed to support the findings of this study.

## Introduction

Heart failure (HF) has been defined as a global pandemic affecting 64.3 million people worldwide [1], and its prevalence is expected to rise due to improved survival with the availability of life-saving evidence-based treatments and the overall longer life expectancy of the general population [2]. HF remains associated with high mortality and morbidity, poor quality of life, and functional capacity and confers a substantial burden on the healthcare system [3]. Studies on the prevalence of HF among populations based in Africa, including Ethiopia, are scarce; however, according to the published hospital prevalence studies, 9.4% to 42.5% of all hospitalizations were related to HF [4].

According to the findings of the OPTIMIZE-HF registry, poor prognostic factors for patients hospitalized with HF were older age, low systolic blood pressure (SBP), low sodium level, increased heart rate, or raised serum creatinine (SCr) at admission [5]. The THESUS-HF registry revealed that predictors of 180-day mortality after an acute HF (AHF) event were malignancy, severe pulmonary disease, smoking, lower SBP, higher heart rate, congestive state, higher blood urea nitrogen (BUN), anemia, and retroviral infection [6]. Though these prognostic factors of HF are well recognized in many studies, it is prudent to investigate additional factors for a comprehensive understanding of HF prognosis.

Multiple electrolyte disorders are often present in patients with HF, with hyponatremia, hypokalemia, and hypochloremia being the most common [7]. Hyponatremia is the most common electrolyte disorder among hospitalized patients with HF [8], and in the OPTIME-CHF and ESCAPE trials, hyponatremia was an independent predictor of mortality in patients with HF [9, 10]. Despite the long-standing focus of guidelines and clinicians on sodium’s role in HF, it has recently been demonstrated that chloride plays a more significant role in the prognosis of HF [11]. Alterations in chloride homeostasis have been associated with a lower glomerular filtration rate, which is a sign of a worse prognosis for patients with HF [12]. Hypochloremia in HF may be related to excess water in the body (hemodilution) as a result of the non-osmotic release of arginine vasopressin, or it may also be related to the depletion of chloride ions, which is frequently due to the administration of loop diuretics [13, 14].

Though there are several studies on HF, the prevalence of hypochloremia and its impact on the outcomes of patients with HF has gotten strong attention in only a few studies. According to the previously published studies, the prevalence of hypochloremia in HF ranged from 10.7% to 31.5% [15–17]. A post hoc analysis of the BEST trial revealed that hypochloremia was strongly associated with an increased risk of mortality among patients with chronic HF, regardless of sodium levels [18]. A study of patients with HF and preserved ejection fraction from the TOPCAT trial found a correlation between low serum chloride levels and an increased risk of HF hospitalization, cardiovascular death, and all-cause mortality [19].

The rationale behind the current study was the emerging need for further research regarding the prevalence, associated factors, and outcomes of hypochloremia in AHF. Besides, such research needs to involve diverse populations, including African patients, due to the potential variations in genetic, environmental, and lifestyle factors that could affect the prevalence and outcomes of hypochloremia in patients with HF. The main purpose of this study was to show the burden and prognostic role of hypochloremia in patients with AHF in Africa, particularly in Ethiopia. Hence, this study aimed to assess the prevalence, associated factors, and prognostic role of hypochloremia in patients with AHF at Yekatit 12 Hospital Medical College (Y12HMC), Addis Ababa, Ethiopia.

## Methods

### Study design

This was a single-center retrospective analysis of AHF patients who had chloride determination on admission and were hospitalized in the medical wards or medical intensive care unit (ICU) of Y12HMC, Addis Ababa, Ethiopia, from July 1, 2022, to July 1, 2023. Y12HMC is one of the largest public teaching hospitals in Addis Ababa, delivering both clinical and academic services. The internal medicine department of Y12HMC has a medical emergency unit, three general medical wards, an isolation ward, and a medical ICU. Patients indicated for hospitalization due to AHF are directly admitted to the medical wards or medical ICU and are managed by a team of nurses, interns, general practitioners, residents, internists, and cardiologists.

### Source and study population

All AHF patients admitted to public hospitals in Addis Ababa, Ethiopia, were considered the source population. All AHF patients, aged ≥15 years, with chloride determination upon admission to the medical wards or medical ICU of Y12HMC during the study period were the study population.

### Eligibility criteria

All AHF patients, aged ≥15 years, with chloride determination upon admission to the medical wards and medical ICU of Y12HMC during the study period were included in the study, whereas those AHF patients with incomplete documentation of essential clinical or laboratory data were excluded from the study.

### Sample size and sampling technique

The minimum sample size was calculated to be 174 using a prevalence of 13% for hypochloremia among hospitalized patients with AHF from a previous study [16], a 95% confidence level (CI), a standard deviation of 0.5, and a margin of error (d) of 5%. The following formula was used to calculate the sample size:

$$\text{n}=\frac{{{\text{Z}}_{1-\alpha /2}}^{2}\times \text{p}\left(1-\text{p}\right)}{{\text{d}}^{2}}=\frac{1.96^{2}\times 0.13\left(1-0.13\right)}{0.05\times 0.05}=174$$

All the AHF patients who fulfilled the eligibility criteria were included in the study with a convenience sampling technique.

### Study variables

The dependent variables were the prevalence of hypochloremia on admission and in-hospital mortality. Admission chloride level was used in this study based on the assumption that it would be available at baseline and that it would have an immediate effect on the outcome of patients with AHF. The independent variables for both of the dependent variables were site of admission, age, sex, comorbidities (atrial fibrillation, hypertension [HTN], ischemic heart disease, diabetes mellitus [DM], chronic kidney disease [CKD]), New York Heart Association (NYHA) classification, SBP, diastolic blood pressure (DBP), sodium, potassium, BUN, SCr, hemoglobin (Hgb), left ventricular ejection fraction (LVEF), and length of hospital stay. Additional independent variables for the prevalence of hypochloremia were use of diuretics prior to presentation (loop diuretics, mineralocorticoid receptor antagonists [MRAs] and thiazide diuretics) and signs of AHF (rales, raised jugular venous pressure, and peripheral edema).

### Data collection procedures

Two general practitioners were trained on data collection, and they extracted and filled out a pretested data abstraction format that was prepared by reviewing similar studies. The sociodemographic variables, clinical profiles, laboratory findings, and outcomes of AHF patients were all collected from the electronic medical records and documented in the data abstraction format. LVEF was taken from the echocardiography report of the patients, and a reduced LVEF was defined when it was ≤ 40%. To ensure the completeness of the data abstraction format, a pre-test was conducted among 30 patients. Proper modifications were employed to the format, including the removal of unavailable variables, including serum calcium and magnesium, and a replacement of non-specific variables with more defined ones, such as changing renal dysfunction to CKD.

### Operational definitions

Hypochloremia was defined by a serum chloride concentration < 96 mEq/L [20].

Hyponatremia was defined by a serum sodium concentration < 135 mEq/L [21].

Hypokalemia was defined by a serum potassium concentration < 3.5 mEq/L [22].

AHF was defined as a clinical syndrome characterized by the development of new HF symptoms (dyspnea, orthopnea, or swelling of the lower extremities) and signs (elevated jugular venous pressure, pulmonary congestion, or peripheral edema), occurring due to decreased cardiac output and/or elevated intracardiac pressures [23].

De novo (new-onset) HF was defined as the development of HF symptoms and signs in patients without a history of HF [23].

Acute decompensated HF was defined as the worsening of HF symptoms and signs in patients with a prior history of HF [23].

HTN was defined by the presence of an average SBP ≥140 mm Hg, DBP ≥90 mm Hg, or self-reported use of antihypertensive medications [24].

Ischemic heart disease (coronary artery disease) was the term given to a cardiac disorder caused by narrowed coronary arteries that supply blood to the heart muscle. Its diagnosis is made based on the presence of angina pectoris, prior myocardial infarction, or a prior history of coronary artery revascularization [25].

DM was defined by a fasting blood glucose level ≥ 126 mg/dL, RBS ≥ 200 mg/dL with symptoms of hyperglycemia, glycated Hgb ≥ 6.5%, or taking medications for DM [26].

CKD was defined as an estimated glomerular filtration rate (GFR) < 60 mL/min/1.73 m2, or the presence of kidney damage (proteinuria, urinary sediment abnormalities, or pathologic or imaging abnormalities) that has been present for at least 3 months [27].

Atrial fibrillation was defined as a supraventricular tachyarrhythmia confirmed by the presence of an arrhythmia on a standard surface electrocardiogram [28].

NYHA classification was used to categorize HF on a scale of I to IV; class I: no limitation of physical activity, class II: slight limitation of physical activity, class III: marked limitation of physical activity, and class IV: occurrence of symptoms even at rest [29].

### Data entry and statistical analysis

Statistical Package for Social Sciences version 26 was used to enter and analyze the data. The sociodemographic data, clinical profiles, laboratory findings, and outcomes of AHF patients, stratified by the presence and absence of hypochloremia, were compiled using descriptive analysis. Continuous variables were expressed as mean (SD) when normally distributed and median (IQR) when not normally distributed. Categorical variables were also summed up as a percentage of the total. The frequencies of categorical variables were compared using Pearson’s chi-square test, whereas the mean and median values of continuous variables were compared using independent samples T test and median test, respectively. Simple and multiple logistic regression analyses were performed to determine factors associated with hypochloremia and to assess the association of hypochloremia and other factors with in-hospital mortality by using the crude odds ratio (COR) and adjusted odds ratio (AOR), respectively, with the accompanying 95% confidence interval (CI). The model assumptions were fulfilled based on the Hosmer-Lemeshow test results. Independent variables with <20% missing values were candidates for simple logistic regression analysis [30], and those with a *P* value <0.25 in the simple logistic regression analysis were subsequently included in the multiple logistic regression analysis, and a two-tailed *P* value <0.05 was regarded as statistically significant [31].

### Ethical approval

Ethical clearance was obtained from the Institutional Review Board (IRB) of Y12HMC (Ref.No.178/13) and the need for consent was waived by the ethics committee as only anonymized patient data was collected retrospectively from electronic medical records. Patients’ data were accessed for research purposes between November 1, 2023, and February 1, 2024.

## Results

### Socio-demographic characteristics and medical history

A total of 267 AHF patients who had a determination of chloride level on admission were found to be eligible and included in this study. Of 267 patients, 92.7% were admitted to the medical wards, and 7.3% were admitted to the medical ICU. The mean age of the patients was 56.7 years [standard deviation (SD): 18.6], and the gender-based distribution of the patients was nearly equal. The most common comorbidity was HTN (48.3%), and the most commonly utilized diuretic agents before presentation to the hospital were the loop diuretics (45.3%). Age, sex, comorbidities, use of diuretics before presentation (loop diuretics, MRAs and thiazide diuretics), NYHA class, and type of AHF were not associated with hypochloremia (Table 1).

**Table 1:** Socio-demographic characteristics and medical history stratified by the presence and absence of hypochloremia at Y12HMC from July 1, 2022, to July 1, 2023.

Characteristics	Overall (n = 267)	Hypochloremia		*P* value*
		Yes (n = 98)	No (n = 169)	
Site of admission				0.542
ICU	21 (7.9)	9 (42.9%)	12 (57.1)	
Medical ward	246 (92.1)	89 (36.2)	157 (63.8)	
Sociodemographic data				
Age (years)–mean (SD)	56.7 (18.6)	59.3 (17.8)	55.2 (19)	0.080
Female, n (%)	138 (51.7)	55 (56.1)	83 (49.1)	0.269
Comorbid conditions				
Atrial fibrillation, n (%)	65 (24.3)	28 (28.6)	37 (21.9)	0.220
Ischemic heart disease, n (%)	54 (20.2)	17 (17.3)	37 (21.9)	0.373
HTN, n (%)	129 (48.3)	43 (43.9)	86 (50.9)	0.269
DM, n (%)	57 (21.3)	21 (21.4)	36 (21.3)	0.855
CKD, n (%)	24 (9)	6 (6.1)	18 (10.7)	0.212
Diuretics (before presentation)				
Loop diuretics, n (%)	121 (45.3)	47 (48)	74 (43.8)	0.509
MRAs, n (%)	53 (19.9)	23 (23.5)	30 (17.8)	0.259
Thiazide diuretics, n (%)	6 (2.2)	3 (3.1)	3 (1.8)	0.494
Category of acute HF				0.062
Acute decompensated HF	149 (55.8)	62 (63.3)	87 (51.5)	
New-onset HF	118 (44.2)	36 (36.7)	82 (48.5)	
NYHA classification				0.095
IV	188 (70.4)	75 (76.5)	113 (66.9)	
II/III	79 (29.6)	23 (23.5)	56 (33.1)	

* The *P* values were obtained using the Pearson’s chi-squared test for the comparison of categorical variables and the independent samples T-test for the comparison of mean age. CKD, chronic kidney disease; DM; diabetes mellitus, HF, heart failure; HTN, hypertension; ICU, intensive care unit; MRAs, mineralocorticoid receptor antagonists; NYHA, New York Heart Classification; SD, standard deviation.

### Clinical and laboratory findings

Of 267 patients, 149 (55.8%) were diagnosed to have acute decompensated HF, and the rest were diagnosed to have de novo (new-onset) HF. The majority of patients (70.4%) had NYHA class IV HF. Overall, 137 (51.3%) of the patients had a SBP < 120 mm Hg and 25 (9.4%) had a DBP < 60 mm Hg, both of which were positively associated with hypochloremia (Table 2). The majority (78.7%) had peripheral edema, 56.9% had rales, and 23.6% had raised JVP, none of which were associated with hypochloremia. A low Hgb value (<12 g/dL) was found in 29.1% of the patients, and this was negatively associated with hypochloremia. Of 133 patients, 47.4% had a raised BUN value, and of 256 patients, 28.9% had a raised SCr value, neither of which was associated with hypochloremia. Of 221 patients, 79 (35.7%) had a reduced LVEF (≤ 40%), which was not associated with hypochloremia (Table 2).

**Table 2:** Physical findings, laboratory values, and outcomes of patients with AHF stratified by the presence and absence of hypochloremia at Y12HMC, July 1, 2022, to July 1, 2023.

Laboratory values	Overall (n = 267)	Hypochloremia		*P* value*
		Yes (n = 98) No. (%)	No (n = 169) No. (%)	
SBP < 120 mm Hg, n (%)	137 (51.3)	66 (67.3)	71 (42)	<0.001
DBP <60 mm Hg, n (%)	25 (9.4)	17 (17.3)	8 (4.7)	0.001
Raised JVP, n (%)	63 (23.6)	26 (26.5)	37 (21.9)	0.390
Rales, n (%)	152 (56.9)	59 (60.2)	93 (55)	0.410
Peripheral edema, n (%)	210 (78.7)	81 (82.7)	129 (76.3)	0.224
LVEF ≤ 40%, no. / total no. (%)	79/221 (35.7)	27 (33.8)	52 (36.9)	0.641
Sodium <135 mEq/L, n (%)	97 (36.3)	73 (74.5)	24 (14.2)	<0.001
Potassium <3.5 mEq/L), n (%)	44 (16.5)	22 (22.4)	22 (13)	0.045
Hgb <12 g/dL, no. / total no. (%)	77/265 (29.1)	19 (19.6)	58 (34.5)	0.010
SCr >1.2 mg/dL, no. / total no. (%)	74/256 (28.9)	28/93 (30.1)	46/163 (28.2)	0.749
BUN > 20mg/dL, no. / total no. (%)	63/133 (47.4)	25 (52.1)	38 (44.7)	0.413
**In-hospital outcomes**				
LOS (days)–median (IQR)	9 (6–14)	10 (7–15)	9 (6–13)	0.045
Death, n (%)	24 (9)	16 (16.3)	8 (4.7)	0.001

* The *P* values were obtained using Pearson’s chi-squared test for comparison of the categorical variables, and the median test for the comparison of LOS. AHF, acute heart failure; BUN, blood urea nitrogen; Cl, chloride; DBP, diastolic blood pressure; Hgb, hemoglobin; IQR, interquartile range; JVP, jugular venous pulse; LOS, length of hospital stay; SBP, systolic blood pressure; SCr, serum creatinine; SD, standard deviation.

### Prevalence of hypochloremia in patients with AHF

**Fig 1:**
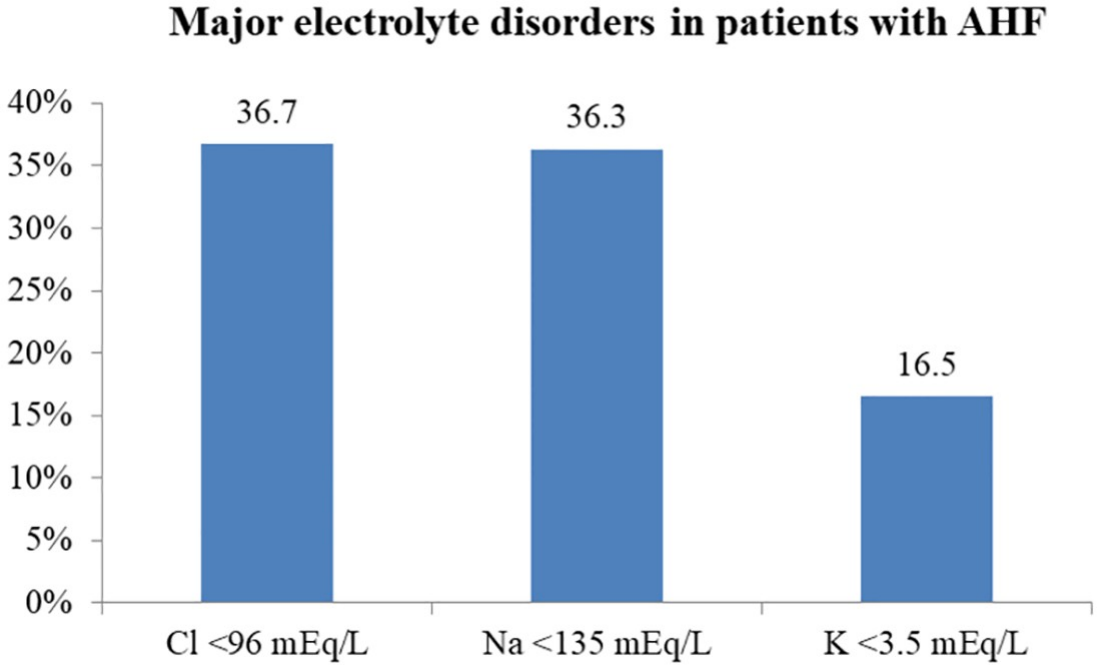
Prevalence of the major electrolyte disorders in patients with AHF at Y12HMC from July 1, 2022, to July 1, 2023. AHF, acute heart failure; Cl, chloride; K, potassium; Na, sodium.

Hypochloremia was observed in 36.7% of hospitalized AHF patients. The prevalence of hypochloremia among AHF patients admitted to the medical wards and medical ICU was 36.2% and 42.9%, respectively, with no significant difference in prevalence based on the site of admission (Table 1). Hyponatremia and hypokalemia were reported in 36.3% and 16.5% of the AHF patients, respectively (Fig 1), and hypochloremia was positively associated with hyponatremia and hypokalemia (Table 1).

### Factors associated with hypochloremia in patients with AHF

In the simple logistic regression analysis, SBP < 120 mm Hg, DBP < 60 mm Hg, hyponatremia, and hypokalemia were significantly associated with hypochloremia. However, in the multiple logistic regression analysis, only low diastolic blood pressure (<60 mm Hg) and hyponatremia were found to be significantly associated with hypochloremia. Patients with low DBP were 3.6 times more likely to have hypochloremia than patients with normal or high DBP (AOR = 3.63, 95% CI, 1.04, 12.72, *P* value = 0.043). Patients with hyponatremia were 29.2 times more likely to develop hypochloremia than those without hyponatremia (AOR = 29.20, 95% CI, 13.21, 64.56, *P* value = <0.001). On the contrary, hypochloremia was negatively associated with low Hgb (<12 g/dL) with an AOR of 0.30 (95% CI, 0.13, 0.71, *P* value = 0.006) (Table 3).

**Table 3:** Simple and multiple logistic regression analysis to assess the factors associated with hypochloremia in patients with AHF at Y12HMC from July 1, 2022, to July 1, 2023.

Characteristics	Simple logistic regression analysis			Multiple logistic regression analysis		
	COR	95% CI	*P* value	AOR	95% CI	*P* value
Age	0.99	(0.98, 1.00)	0.081	0.98	(0.96, 1.00)	0.112
Atrial fibrillation	1.43	(0.81, 2.52)	0.222	1.22	(0.53, 2.82)	0.644
CKD	0.55	(0.21, 1.43)	0.218	0.95	(0.25, 3.62)	0.938
Type of HF						
Acute decompensated HF	1.62	(0.98, 2.70)	0.062	1.05	(0.51, 2.17)	0.899
New-onset HF	1.0			1.0		
NYHA classification of HF						
IV	1.62	(0.92, 2.85)	0.097	2.08	(0.90, 4.82)	0.086
II/III	1.0			1.0		
SBP <120 mm Hg	2.85	(1.69, 4.79)	<0.001	1.92	(0.93, 3.99)	0.079
DBP <60 mm Hg	4.22	(1.75, 10.20)	0.001	3.63	(1.04, 12.72)	0.043
Peripheral edema	1.48	(0.79, 2.78)	0.226	1.61	(0.69, 3.79)	0.272
Sodium <135 mEq/L	17.64	(9.43, 33.02)	<0.001	29.20	(13.21, 64.56)	<0.001
Potassium <3.5 mEq/L	1.93	(1.01, 3.72)	0.048	2.23	(0.89, 5.57)	0.086
Hgb <12 g/dL	0.462	(0.26, 0.84)	0.011	0.30	(0.13, 0.71)	0.006

AHF, acute heart failure; CKD, chronic kidney disease; DBP, diastolic blood pressure; HF, heart failure; Hgb, hemoglobin, NYHA, New York Heart Association; SBP, systolic blood pressure.

### Outcomes of hypochloremia in patients with AHF

The median length of hospital stay (LOS) for patients with AHF was 9 days (IQR: 6–14). The LOS was significantly longer in patients with hypochloremia (10 days) as compared with those without hypochloremia (9 days). The overall in-hospital mortality rate of AHF patients was 9%, but the mortality rate in patients with hypochloremia (16.3%) was significantly higher than that of the patients without hypochloremia (4.7%) (Table 2). A simple logistic regression analysis revealed a strong association between hypochloremia and in-hospital mortality of AHF patients (COR = 3.92, 95% CI: 1.61, 9.56, *P* value = 0.003). This finding remained significant after adjusting for ICU admission, SBP < 120 mm Hg, and DBP < 60 mm Hg in the multiple logistic regression analysis, where the odds of in-hospital mortality among AHF patients with hypochloremia were 2.8 times higher compared to patients without hypochloremia (AOR = 2.82, 95% CI: 1.08, 7.04, *P* value = 0.035). However, the other electrolyte disorders (hyponatremia and hypokalemia) didn’t have a significant association with mortality in patients with AHF (Table 4).

**Table 4:** Simple and multiple logistic regression analysis to assess the association of hypochloremia and other factors with in-hospital mortality in patients with AHF at Y12HMC from July 1, 2022, to July 1, 2023.

Variables	Simple logistic regression analysis			Multiple logistic regression analysis		
		95% CI	*P* value	AOR	95% CI	*P* value
ICU admission	6.74	(2.40, 18.91)	<0.001	5.86	(1.91, 17.96)	0.002
SBP <120 mm Hg	5.39	(1.79, 16.22)	0.003	3.28	(1.02, 10.51)	0.045
DBP <60 mm Hg	3.93	(1.40, 11.07)	0.010	2.12	(0.67, 6.65)	0.199
Chloride <96 mEq/L	3.92	(1.61, 9.56)	0.003	2.82	(1.08, 7.04)	0.035
Sodium <135 mEq/L	1.55	(0.66, 3.60)	0.313			
Potassium <3.5 mEq/L	1.38	(0.49, 3.91)	0.548			

AHF, acute heart failure; DBP, diastolic blood pressure; ICU, intensive care unit; SBP, systolic blood pressure.

## Discussions

This study, the first of its kind in sub-Saharan Africa, has assessed the prevalence, associated factors, and prognostic role of hypochloremia in patients with AHF at a single center in Ethiopia. It has revealed a high prevalence of hypochloremia in patients with AHF, and it has shown that low diastolic blood pressure (<60 mm Hg) and hyponatremia were significantly associated with the development of hypochloremia. It also found that the in-hospital mortality in AHF patients with hypochloremia was significantly higher than that of patients without hypochloremia, irrespective of blood pressure values and site of admission to the medical wards or medical ICU.

There have been only a few published studies that assessed the prevalence of hypochloremia in patients with AHF. In this study, hypochloremia was the most common electrolyte disorder reported in 36.7% of AHF patients. In contrast, in a study done in tertiary care hospitals in Ethiopia, the commonest electrolyte abnormality was hyponatremia (43%), but chloride disorder was not reported [32]. The prevalence of hypochloremia in the current study was higher than that of the PROTECT study (13%) [16] and a cohort study on HF (31.5%) [17]. Besides, the prevalence of hypochloremia in the current study was also higher than observed in another study involving chronic HF patients (10.7%) [15]. These disparities in the prevalence of hypochloremia in HF patients might be explained by the variations in the underlying pathophysiologic mechanisms of hypochloremia in HF, including the degree of congestion due to HF, use of medications like loop diuretics, and the amount of dietary sodium chloride intake [13, 14, 33].

This study revealed that DBP <60 mm Hg and hyponatremia were significantly associated with hypochloremia, and likewise, lower DBP and lower sodium values were also significantly associated with lower chloride levels in patients hospitalized for AHF in the PROTECT trial [16]. Consistent with the current study, low admission chloride level was also positively associated with hyponatremia in the ROSE-AHF trial, which included 360 patients with AHF [34]. This underscores the importance of paying attention to chloride values when AHF patients present with low blood pressure and sodium values.

It is well recognized that loop and thiazide diuretics can potentially deplete serum chloride levels mainly by preventing reabsorption at the ascending loop of Henle and the distal convoluted tubule [35–37]. However, there was no significant association between prior utilization of diuretics and hypochloremia in the current study, implying that the occurrence of hypochloremia might not be mainly driven by chloride depletion due to diuretic therapy. On the other hand, the strong association of hypochloremia with hyponatremia in the current study supports the possibility of hemodilution as the predominant contributor to the development of hypochloremia in patients with AHF as opposed to chloride depletion due to diuretic therapy, which is expected to result in hypochloremia with normal sodium values [15].

The current study revealed that the median length of hospital stay was significantly longer in AHF patients with hypochloremia (10 days) compared to those without hypochloremia (9 days). Similar results have been found in a study of 1,318 consecutive patients with chronic HF admitted for acute decompensated HF to the Cleveland Clinic, where the median length of hospital stay was longer in patients with a Cl level <99 mEq/L (9.7 days) than in patients with a Cl level between 99 and 103 mEq/L (9 days) and >103 mEq/L (7.9 days) [19]. These findings imply that the prevention or correction of hypochloremia may help shorten the hospitalization period of acute HF patients.

Though chloride is a neglected electrolyte in patients with HF, it has been implicated as a possible cause of grave prognosis in patients with AHF [38]. The current study showed that the in-hospital mortality rate of AHF patients with hypochloremia (16.3%) was significantly higher than that of patients without hypochloremia (4.7%). The association of hypochloremia with poor outcomes in HF patients was also observed in other studies. According to the analysis of the TOPCAT trial, which studied patients with HF with preserved ejection fraction, there was an increased risk of HF hospitalization, cardiovascular death, and all-cause mortality [19]. Similarly, the PROTECT study has shown that patients with AHF who developed new or chronic hypochloremia 14 days later were less likely to survive [16]. Furthermore, a post hoc analysis of the BEST trial revealed a strong association between hypochloremia and increased risk of mortality among chronic HF patients, independent of sodium levels [18].

In the current study, there was no significant difference in the prevalence of hypochloremia among AHF patients admitted to the medical wards and the medical ICU. On the other hand, the association between hypochloremia and in-hospital mortality was significant before and after the adjustment of important confounders, including ICU admission. However, there have not been similar studies that compared the prevalence of hypochloremia in HF patients based on their site of admission. One study on HF patients admitted to the ICU in a medical center in Boston, Massachusetts, showed a lower prevalence of hypochloremia (9.3%) compared to the current study and revealed that those patients with hypochloremia had significantly higher in-hospital mortality than those with normal chloride levels [39].

This study has some limitations. Firstly, due to the retrospective nature of this study, the findings are dependent on the availability and accuracy of the medical records. Secondly, the results may not be generalizable to all AHF patients, particularly those admitted to the ICU, due to the small sample size and inclusion of patients from a single center. Finally, careful interpretation of the findings is crucial, as definitive causation between hypochloremia and associated factors or outcome variables cannot be established due to the design of the study.

## Conclusions

This study revealed a high prevalence of hypochloremia among patients with AHF. Low diastolic blood pressure and hyponatremia were significantly associated with the development of hypochloremia. Most importantly, AHF patients with hypochloremia had a higher in-hospital mortality rate compared to those without hypochloremia. Hence, hypochloremia on admission should be considered a potential prognostic factor in patients with AHF, and further research with a larger sample size is needed to support the findings of this study.

## Supporting information

S1 Checklist Human participants research checklist. (DOCX)

S2 Checklist STROBE statement: Checklist of items that should be included in reports of observational studies. (DOCX)

S1 Data (SAV)

## List of abbreviations

AHF: Acute heart failure
BUN: Blood urea nitrogen
CKD: Chronic kidney disease
Cl: Chloride
DBP: Diastolic blood pressure
DM: Diabetes mellitus
HF: Heart Failure
Hgb: Hemoglobin
HTN: Hypertension
ICU: Intensive care unit
IQR: Interquartile range
LOS: Length of hospital stay
LVEF: Left ventricular ejection fraction
MRAs: Mineralocorticoid receptor antagonists
NYHA: New York Heart Association
SBP: Systolic blood pressure
SCr: Serum creatinine
SD: Standard deviation
Y12HMC: Yekatit 12 Hospital Medical College
