# Supplementary material for: Prevalence and prognostic role of hypochloremia in patients with acute heart failure in Ethiopia: A single-center retrospective analysis
Source: PLoS One. 2024 Sep 12;19(9):e0310251. doi: 10.1371/journal.pone.0310251 (PMC11392231; doi:10.1371/journal.pone.0310251)
Supplement: S2 Checklist — (DOCX) [file pone.0310251.s002.docx]

**STROBE Statement: Checklist of items that should be included in reports of observational studies**

|  | **Item**  **No** | **Recommendation** |
| --- | --- | --- |
| **Title and abstract** | 1 | Study design was stated in the title  An informative and balanced summary of what was done and what was found is provided in the abstract. |
|  |  |  |
| **Introduction** |  |  |
| Background/rationale | 2 | The scientific background and rationale for the investigation being reported was explained |
| Objectives | 3 | Specific objectives were stated |
| **Methods** |  |  |
| Study design | 4 | Key elements of study design are presented early in the paper |
| Setting | 5 | The setting, locations, and relevant dates, and data collection were described |
| Participants | 6 | The eligibility criteria, and the sources and methods of selection of participants were stated. |
|  |  |  |
| Variables | 7 | The outcomes and all the potential predictors and confounders are clearly defined. |
| Data sources/ measurement | 8* | Sources of data and details of methods of assessment (measurement) were given for each variable of interest |
| Bias | 9 | Efforts to address potential sources of bias were not described but it was reported as a limitation |
| Study size | 10 | The study size determination was explained |
| Quantitative variables | 11 | The way of handling quantitative variables in the analyses was explained |
| Statistical methods | 12 | All the statistical methods used in the study were explained. |
|  |  | Methods used to examine subgroups and interactions were described. |
|  |  | Management of missing data was addressed |
|  |  | The analytical methods were described. |
|  |  |  |
|  |  |  |

| **Results** |  | |
| --- | --- | --- |
| Participants | 13 | 1. The number of individuals included in the study is described |
|  |  | 1. Reasons for non-participation were described as exclusion criteria |
|  |  |  |
| Descriptive  data | 14 | Characteristics of study participants were given |
| Outcome data | 15 | Numbers of outcome events are reported. |
|  |  |  |
|  |  |  |
| Main results | 16 | (*a*) Unadjusted estimates and confounder-adjusted estimates and their precision were given. |
|  |  | (*b*) Category boundaries were reported when continuous variables were categorized |
|  |  |  |
| **Discussion** |  |  |
| Key results | 18 | Key results with reference to study objectives were summarized |
| Limitations | 19 | Limitations of the study were discussed. |
| Interpretation | 20 | Overall interpretation of results considering objectives, limitations, multiplicity of analyses, results from similar studies, and other relevant evidence was done cautiously. |
| Generalizability | 21 | The generalizability (external validity) of the study results was discussed as a limitation |

**Other information**

Funding 22 The absence of funding for this study was described.
